# Extraction, Structures, Bioactivities and Structure-Function Analysis of the Polysaccharides From Safflower (*Carthamus tinctorius* L.)

**DOI:** 10.3389/fphar.2021.767947

**Published:** 2021-10-20

**Authors:** Xiaoyi Wu, Xinbo Cai, Jiaxuan Ai, Chi Zhang, Nan Liu, Wei Gao

**Affiliations:** ^1^ School of Traditional Chinese Medicine, Capital Medical University, Beijing, China; ^2^ Beijing Shijitan Hospital, Capital Medical University, Beijing, China

**Keywords:** safflower (*Carthamus tinctorius* L.), polysaccharide, structure, bioactivity, structure-bioactivity relationship

## Abstract

Safflower (*Carthamus tinctorius* L.) is a herbal plant with a long history of clinical application worldwide, such as coronary heart disease, hypertension, dysmenorrhea and amenorrhea. It is also extensively used as an important oilseed plant for hundreds of years in some countries, like China, India, Mexico and the United States. Therefore, safflower is believed as a crop with dual values of medicine and economy as well. Safflower polysaccharides (SPS), from the plant, are believed as one of the most important biologically active components with multiple pharmacological properties, including anti-tumor, immune regulation, anti-oxidation, and anti-cerebral ischemia reperfusion injury effects. The polysaccharides, from bee pollen of safflower, named PBPC, also attract the attention of researchers because of their particular origin and bioactivities. Although the extraction, purification, structure and biological activities of SPS and PBPC have been studied for decades, there is not any available review both concerning SPS and PBPC. In this condition, this paper aims to systematically review the research progress in extraction, purification, structural characteristics, and bioactivities of SPS and PBPC, and provide basis for the in-depth study about their structure-bioactivity relationship. It will serve as a methodological outline for further research in fields of new drug discovery and clinical application of SPS or PBPC, and simultaneously remind us of unresolved problems noted in the polysaccharide research.

## 1 Introduction

Safflower (*Carthamus tinctorius* L.) is a medicinal herb with a long history of clinical use for conditions such as coronary heart disease, hypertension, dysmenorrhea and amenorrhea (Delshad et al., 2018). Safflower has been cultivated in more than 60 countries all over the world. In the Chinese medicine theory, safflower is a traditional medicine to activate blood circulation and remove blood stasis (Yao et al., 2016). Modern pharmacological studies have shown that safflower has many beneficial bioactivities, such as anti-inflammatory (Zhang and Zheng, 2010), antioxidant (Wu et al., 2021), and antitumor effects (Wang et al., 2016), protective effects against cerebral ischemia injury (Liu F. et al., 2018), and ameliorating myocardial ischemia (Ye et al., 2020a). Safflower-related preparations have been widely applied in clinic, such as safflower injection (Wang et al., 2018), safflower yellow injection (Li et al., 2015) and safflower soothing and activating collaterals liniment (Niu et al., 2018). In addition, safflower seed has been extensively used as an important oilseed for hundreds of years in a number of countries, including China, India, Mexico and the United States (Wu et al., 1982). Therefore, safflower is considered a dual crop with combined medicinal and economic value (Yang Y. X. et al., 2004).

**FIGURE 1:**
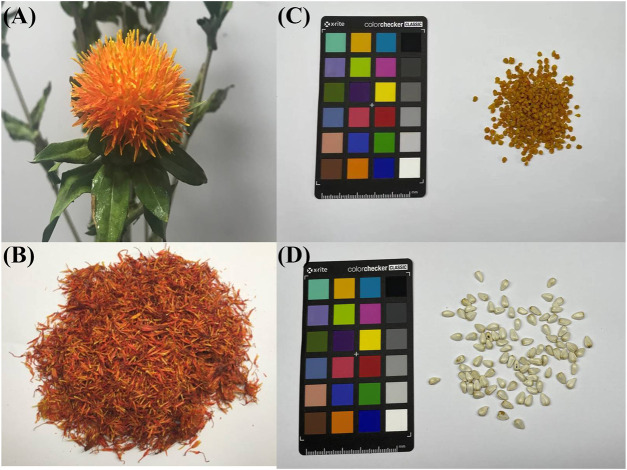
The flower, bee pollen and seeds of safflower [**(A)**: fresh flower, **(B)**: dried flower, **(C)**: bee pollen, **(D)**: seeds).

The main components of safflower are polysaccharides, flavonoids, volatile oils, alkaloids, lignans and organic acids (Sato et al., 1985; Zhou et al., 1995; Ren R. T. et al., 2012; Ren et al., 2013; Hong et al., 2015; Lin et al., 2018; Li X. R. et al., 2021). Safflower polysaccharides (SPS) are water-soluble heteropolysaccharides extracted from the flower of safflower, with multiple pharmacological properties, including anti-tumor, immunoregulatory, anti-oxidative, and anti-coagulant effects, which were found to be protective against cerebral ischemia reperfusion injury and steroid induced avascular necrosis of the femoral head (Zhou et al., 2008; Zhang and Zheng, 2010; Tao et al., 2011; Ren et al., 2016; Wan, 2016; Cui et al., 2020). Owing to the nontoxicity and negligible side effects of natural polysaccharides, SPS are considered potential drug candidates for preventing and treating various diseases (Yao, 2019). Currently, SPS and their bioactivities have gained growing attention from scholars around the world. Additionally, polysaccharides from the bee pollen collected from *Carthamus tinctorius* (PBPC) have also attracted the attention of scholars because of their particular origin and bioactivities. Bee pollen of safflower is a mixture of the pollen and nectar of safflower and bee gland secretions, which not only supplies nutrition to bees and larvae but is also popular as a nutritional product (Kocot et al., 2018). Polysaccharides are the most abundant components of bee pollen, accounting for 18.9–57.6% of the dried powder (Li et al., 2019). In recent years, an increasing number of studies have shown that PBPC possess a variety of biological effects, such as antioxidant, immunomodulatory, antitumor, anticoagulant and antibacterial activities (Li et al., 2017c; Zuo et al., 2017; Wang et al., 2019b; Chen et al., 2019; Wang et al., 2019c; Shi et al., 2020). Because the structure and biological activity of bee pollen polysaccharides vary widely depending on the plant source of the pollen, origin and ecological environment, the study of PBPC is significant for its better utilization. Photographs of the plant, flower, seeds and bee pollen of safflower are shown in Figure 1.

From the chemical point of view, natural polysaccharides are complex and diverse. The polysaccharides from different plants are obviously different in aspects of molecular weight, monosaccharide types and glycosidic bonds, and can vary considerably even when collected from the same plant (Yang L. Q. et al., 2004; Yu et al., 2018; Liu et al., 2021). It was also shown that the bioactivities of polysaccharides are determined by their structures (Huang et al., 2020). Thus, developing methods for the preparation of purified SPS and PBPC as well as systematic studies of their structures are necessary. Although the extraction, purification, structure and biological activities of SPS and PBPC have been studied for decades, and some studies have elucidated their main constituent monosaccharide and typical glycosidic linkages (Wakabayashi et al., 1997; Zou, 2011; Yao et al., 2018; Hu, 2020), there is no systematic review analyzing both SPS and PBPC. There is also a lack of research on the structure-bioactivity relationships. Therefore, the aim of this article was to systematically review the research progress in the extraction, purification, structural characteristics, and bioactivities of SPS and PBPC, providing a detailed reference for the study of their structure-bioactivity relationship. It attempts to provide a methodological outline for further research in fields of drug discovery and clinical application of SPS or PBPC, and simultaneously reminds us of unresolved problems noted in the field of natural polysaccharides research.

## 2 Extraction and Purification Methods

### 2.1 Extraction and Purification of Safflower Polysaccharides

**FIGURE 2:**
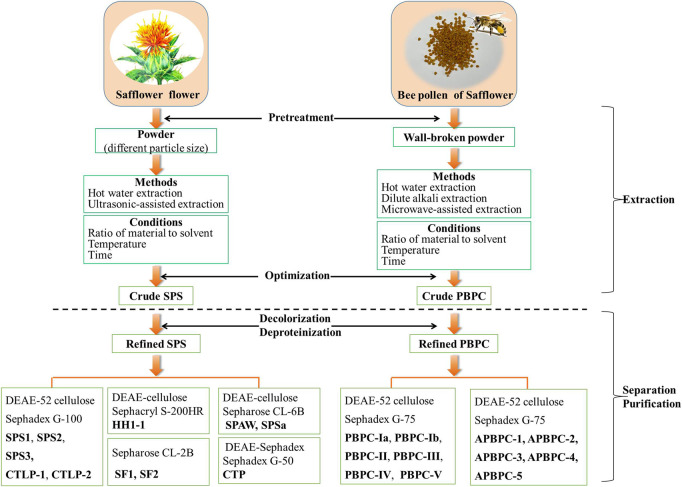
Schematic representation of the extraction and purification of polysaccharides from the flower and bee pollen of safflower (SPS: Safflower polysaccharides; PBPC: Polysaccharides of safflower bee pollen).

Since plant polysaccharides are considered a structural constituent of the cell wall, the appropriate extraction methods for botanical polysaccharides require efficiently breaking the cell wall and accelerating the release of intracellular polysaccharides (Lin et al., 2017). Classical hot water extraction is the convenient approach for the extraction of polysaccharides (Zhang et al., 2010a; Xu et al., 2012), but in addition to this traditional method, other methods for the extraction of SPS have also been reported in recent years (Yang et al., 2014; Yuan et al., 2019). Generally, the dried flowers of safflower are ground into a powder before extraction with distilled water, either as a decoction or ultrasonically. After that, the extraction solutions are centrifuged, concentrated, and precipitated with ethanol to obtain crude SPS. Subsequently, the crude SPS is deproteinized with Sevag reagent, followed by successive washes with ethanol, acetone and ether to remove the fat-soluble ingredients. The final refined SPS can then be obtained by drying the separated precipitate (Xu et al., 2012). The extraction process of SPS is schematically shown in Figure 2. A review of the literature indicates that the yield of crude SPS varies widely under different extraction conditions, ranging from 2.75 to 20.35% (Zhang et al., 2010a; Wang and Wang, 2010; Zou, 2011; Xu et al., 2012; Yang et al., 2012; Deng et al., 2013; Yang et al., 2014; Yuan et al., 2019; Hu, 2020; Wang R. et al., 2021). The detailed information of various methods for the extraction of SPS is listed in Table 1.

**TABLE 1:** Detailed information of various methods for SPS extraction.

No.	Extraction method	Extraction conditions	SPS Yield (%)	References
1	Water extraction	Water/raw material: 22.04/1	2.79	Deng et al. (2013)
		Time: 1.67 h		
		Temperature: 94.71°C		
		2 rounds of extraction		
2	Water extraction	Water/raw material: 24/1	4.86	Zou (2011)
		Time: 1.7 h		
		Temperature: 95°C		
		2 rounds of extraction		
3	Water extraction	Water/raw material: 33/1	6.01	Zhang et al. (2010a)
		Time: 1 h		
		Temperature: 100°C		
		4 rounds of extraction		
4	Water extraction	Water/raw material: 16.69/1	7.45	Wang R. et al. (2021)
		Time: 89.78 min		
		Temperature: 91.39°C		
		3 rounds of extraction		
5	Water extraction	Water/raw material: 30/1	7.50	Xu et al. (2012)
		Time: 1 h		
		Temperature: 100°C		
		4 rounds of extraction		
6	Water extraction	Water/raw material: 20/1	10.19	Wang and Wang (2010)
		Time: 1.5 h		
		Temperature: 80°C		
		3 rounds of extraction		
7	Ultrasonic-assistant extraction	Water/raw material: 25/1	14.16	Yuan et al. (2019)
		Time: 60 min		
		Temperature: 69°C		
		Ultrasonic power: 630 W		
8	Ultrasonic-assistant extraction	Water/raw material: 15/1	16.97	Hu (2020)
		Time: 50 min		
		Temperature: 65°C		
		Ultrasonic power: 135 W		
9	Ultrasonic-assistant extraction	Water/raw material: 25/1	20.35	Yang et al. (2012)
		Time: 55 min		
		Temperature: 65°C		
		Ultrasonic power: 80%		
10	Supermicro-pulverization Ultrasonic-assistant extraction	Water/raw material: 40/1	7.73	Yang et al. (2014)
		Time: 120 min		
		Temperature: 65°C		
		Supermicro-pulverization powder: 200 sieve		
		Ultrasonic power: 600 W		

Extraction conditions including particle size of the raw material powder, extraction method, ratio of raw material to solvent, extraction temperature and time all have great influence on the extraction rate of SPS. Yang et al. (2014) compared the extraction efficiency of safflower powder with different particle sizes, and found that the SPS yield increased 25% when using superfine powder with a particle size of 5–10 μm. The yield of SPS can be improved by different methods. Wang et al. investigated the effects of the solid-liquid ratio, temperature, and time in single factor and orthogonal experiments, which finally resulted in the highest yield of SPS based on hot water decoction reported to date. Under the optimized conditions, including an extraction time of 1.5 h, extraction temperature of 80°C, water: material ratio of 20:1, and three rounds of extraction, the yield of SPS reached up to 10.19% (Wang and Wang, 2010). In a different approach, it was found that ultrasonic-assisted extraction, which has the advantages of convenient operation, short extraction time, and low cost, can break the cell walls more effectively and increase the yield of SPS compared to other methods (Yang et al., 2012; Yuan et al., 2019). The optimal conditions for ultrasonic-assisted extraction were found to encompass a water/raw material ratio of 25:1 (V:m), temperature of 65°C, and ultrasonication time of 55 min, which resulted in a yield of 20.35% (Yang et al., 2012). These various extraction methods are all dedicated to improving the extraction efficiency of SPS, and aim to establish an economical and environmental strategy.

Natural polysaccharides often contain a lot of pigments and impurities, which not only reduces the purity of polysaccharide, but also affects the qualitative and quantitative analysis (Zheng et al., 2017). Therefore, efficient decolorization and purification of SPS is essential. After extraction, the crude SPS can be further purified by a combination of techniques, including deproteinization, decolorization, ion-exchange chromatography, and gel filtration chromatography, as shown in Figure 2 (Zou, 2011; Ren et al., 2013; Hu, 2020). Zou et al. investigated various approaches of the deproteinization and decolorization of SPS, and the optimized method based on a combined enzyme-Sevag method, and HPD-100 macroporous adsorption resin afforded an optimal decolorization rate and retention rate of SPS (Zou et al., 2011). The refined SPS was further separated and purified mainly via ion-exchange chromatography (DEAE-Sepharose, DEAE-Cellulose, DEAE-Sephadex) and gel filtration chromatography (Sephadex G, Sephacryl S, Sepharose CL) (Zhu et al., 2018). Five polysaccharide fractions (**SPS1**, **SPS2**, **SPS3**, **CTLP1**, **CTLP2**) were isolated and purified using a DEAE-52 cellulose column and Sephadex G-100 column (Zou, 2011; Ren et al., 2013; Hu, 2020). After purification on a DEAE-Sephadex A-25 column and size-exclusion column (Sephadex G-50), a single polysaccharide fraction named **CTP** was obtained (Huo et al., 2005a). The purification of safflower polysaccharides (**SF1** and **SF2**) was directly conducted using a Sepharose CL-2B column (Wakabayashi et al., 1997). DEAE cellulose column chromatography and a Sephacryl S-200HR column were used to obtain the polysaccharide fraction **HH1-1** (Yao et al., 2018). **SPAW** and **SPSa** were isolated and purified using a DEAE-cellulose column and a Sepharose CL-6B column (Cui et al., 2019; Cui et al., 2020). Appropriate separation and purification methods should be chosen according to the characteristics of the polysaccharides, while optimizing the convenience and efficiency. Up to now, there is no report on the industrialized production of safflower polysaccharides.

### 2.2 Extraction and Purification of PBPC

There are three main approaches for the extraction of PBPC, including water extraction, dilute alkali extraction and microwave-assisted methods (Zuo and Qian, 2012; Zuo and Qian, 2013; Li et al., 2017c; Shi et al., 2020). Interestingly, traditional hot water extraction remains the method with the best extraction efficiency to date. A schematic representation of the extraction methods of PBPC is shown in Figure 2. In these approaches, the walls of safflower bee pollen are broken and the material is refluxed with petroleum ether (2 h) and 95% ethanol (two times) successively. After filtration, the residues are dried and extracted at 80°C over 12 h with hot water (1:20, w/v) for the first time, and then at the same temperature over 8 h with hot water (1:10, w/v) for the second time. The hot water extraction solutions are then centrifuged, concentrated, and precipitated with ethanol. The resulting precipitate is further processed through a series of treatments including deproteinization with Sevag reagent, followed by washing with ethanol, acetone and ether (three times) to finally obtain the crude PBPC (Zuo and Qian, 2012; Zuo and Qian, 2013). Li et al. (2017b) investigated various approaches for the deproteinization of PBPC using response surface methodolog, and the optimized method was enzyme-Sevag treatment. A DEAE-52 cellulose column and Sephadex G-75 column were used for the separation and purification of polysaccharides, which yielded six polysaccharide fractions designated as **PBPC-Ia**, **PBPC-Ib**, **PBPC-II**, **PBPC-III**, **PBPC-IV**, and **PBPC-V** (Li et al., 2020).

Unlike water extraction, dilute alkali extraction is better for isolating the cell wall bound or intracellular polysaccharides, which may have better antioxidant and free radical scavenging activities (Jiao et al., 2015). Compared with the preparation of water-soluble polysaccharides, alkali-soluble polysaccharide preparation includes an additional extraction with 0.05 M NaOH after water extraction. The alkali-soluble polysaccharides from bee pollen of safflower was then purified using a Sephadex G-75 column and named APBPC, which was further separated on a DEAE-52 cellulose column into the five fractions **APBPC-1**, **APBPC-2**, **APBPC-3**, **APBPC-4**, and **APBPC-5** (Shi, 2021). Compared with these traditional approaches, there was no obvious advantage of microwave-assisted methods (Li et al., 2017c).

## 3 Physiochemical and Structural Features of Safflower Polysaccharides

The structural characteristics of the obtained natural polysaccharides are closely related to the extraction and isolation methods employed, and also determine their biological activity to a certain extent (Zhang et al., 2019). Previous studies had shown that the structures of SPS and PBPC are characterized by diversity and complexity. Thus, it is particularly important to clarify the structural characteristics of polysaccharides by studying their molecular weight (Mw), monosaccharide composition, glycosidic bond type and linkage order of monomeric polysaccharides. The analysis methods for identifying the structure of polysaccharides mainly include partial acid hydrolysis, periodate oxidation, Smith degradation, methylation analysis, as well as chromatographic, spectroscopic and mass spectrometric methods (Huo, 2005; Zou, 2011; Hu, 2020). To date, 11 polysaccharide fractions have been separated and identified from the flowers of safflower, and 11 polysaccharides have been obtained from bee pollen collected from safflower. However, some polysaccharide fractions, such as **SPS1**, **PBPC-Ia**, **PBPC-Ib**, **PBPC-III**, **PBPC-IV**, **PBPC-V**, **APBPC-1**, **APBPC-3**, **APBPC-4** and **APBPC-5** have low abundance, and they lack structural studies due to the unavailability of sufficient material (Zou, 2011; Li et al., 2017a; Shi, 2021). The sources and structural features (Mw, monosaccharide composition and chemical structures) of SPS and PBPC are listed in Tables 2, 3. Notably, the structures of the obtained safflower polysaccharides were still primary structures.

**TABLE 2:** The polysaccharides isolated from flower of safflower: chemical structure.

No.	Compound name	Molecular weight (Da)	Monosaccharide composition	Primary structural features	References
1	SPS1	—	—	—	Ren et al. (2013)
2	SPS2	9.332 × 10^3^	Rha, Ara, Xyl, Man, Glc, Gal in the ratio of 4.44:1.46:4.51:5.82:8.23:19.38	L-(+)-(1→3)-Ara residues, β-D-(1→2)- or β-D-(1→6)- or β-D-(1→3)-Man residues, β-D-(1→2)- or β-D-(1→6)- or β-D-(1→3)-Glc residues, β-D-(1→2)- or β-D-(1→6)- or β-D-(1→3)- Gal residues	Zou (2011); Ren et al. (2013)
3	SPS3	5.861× 10^3^	Rha, Ara, Glc, Gal in the ratio of 2.93:11.19:33.68:3.48	α-L-(+)-(1→2)- or α-L-(+)-(1→3)-Ara residues, α-D-(1→3)-Glc residues, α-D-(1→2)- or α-D-(1→6)- or α-D-(1→3)-Gal residues	Zou (2011); Ren et al. (2013)
4	CTP	4–5 × 10^3^	Glc, Gal in the ratio of 6.08:1	(1→4)-linked-Glc as the main chain with (1→3,6), (1→6), (1→)-linked-Gal, (1→2)-linked-Glc, (1→6), (1→)-linked-Glc, and two (1→6)-linked-Glc	Huo et al. (2005b)
5	CTLP-1	5.9 × 10^3^	Ara, Glc, Gal in the ratio of 6.7:4.2:1	→1)-α-GalA*p*-(1→4)-α-Ara*p*-(1→2)-α-Glc*p*-(4→	Hu, (2020)
6	CTLP-2	8.2 × 10^3^	Ara, Glc, Gal in the ratio of 16.76:4.28:1	→1)-α-Gal*p*-(2,6→1)-α-Ara*p*-(4,6→1)-α- Glc*p*-(3→	Hu, (2020)
7	HH1-1	7.09 × 10^4^	Gal, Ara in the ratio of 54.9:45.1	1,6-linked Gal*p* as the main chain with the branch (1→3)-linked- Gal*p* at C-3, and the sub-branches consisted of T- or 1,5- or 1,3,5-linked Ara residues and T- linked Gal residues	Yao et al. (2018)
8	SPSa	7.1 × 10^4^	Glc	The repeating unit of 1,4,6-β-Glc*p* as the main chain, branched with T-β-Glc*p* at C6 in the molar ratio of 1:1	Cui et al. (2019)
9	SPAW	7.8 × 10^4^	Glc	The repeating unit of (1→3)-linked β-D-Glc*p*	Cui et al. (2020)
10	SF1	>1.0 × 10^5^	Rha, Ara, Xyl, Man, Glc, Gal in the ratio of 2.9:7.5:3.8:1:11.6:8.9	—	Wakabayashi et al. (1997)
11	SF2	>1.0 × 10^5^	Rha, Ara, Xyl, Man, Glc, Gal in the ratio of 2.9:10.3:4.2:1:5.1:8.5	—	Wakabayashi et al. (1997)

Note: “—” represents not studied.

**TABLE 3:** The polysaccharides isolated from bee pollen of safflower: chemical structure.

No.	Compound name	Molecular weight (Da)	Monosaccharide composition	Primary structural features	References
1	PBPC-Ia	5.3 ×10^3^	—	—	Li et al. (2017a)
2	PBPC-Ib	2.575 ×10^5^	—	—	Li et al. (2017a)
3	PBPC-II	3.77 ×10^3^	Rha, Ara, Fuc, Xyl, Glc, Gal in the ratio of 1.40:1.53:1:1.11:2.79:9.73 (Contains uronic acid)	—	Li et al. (2017a); Wang et al. (2019c)
4	PBPC-III	2.636 ×10^4^	—	—	Li et al. (2017a)
5	PBPC-IV	1.171 ×10^5^	—	—	Li et al. (2017a)
6	PBPC-V	1.544 ×10^4^	—	—	Li et al. (2017a)
7	APBPC-1	—	—	—	Shi (2021)
8	APBPC-2	1.6524 ×10^5^	Rha, GluUA, Glc, Gal, Ara in the ratio of 11.93:10.06:13.37:10.29:8.79	Only the α-and β-comfigurations was conducted	Shi (2021)
9	APBPC-3	—	—	—	Shi (2021)
10	APBPC-4	—	—	—	Shi (2021)
11	APBPC-5	—	—	—	Shi (2021)

Note: “—” represents not studied.

### 3.1 Average Molecular Weight

The average Mw of polysaccharides is mainly determined by high performance liquid chromatography (HPLC), gel chromatography, high performance size-exclusion chromatography or high performance gel permeation chromatography (HPGPC) (Huo, 2005; Zou, 2011; Hu, 2020; Shi, 2021). The Mw of isolated SPS ranges from 4 × 10^3^ Da (**CTP**) to >100 × 10^3^ Da (**SF1** and **SF2)**. The Mws of SPS derived from the same crude polysaccharide mixtures were in the same order of magnitude, but an analysis of PBPC from the same source on a Sephadex G-150 gel column, revealed a large variation in Mw, ranging between 3.77 × 10^3^ Da (**PBPC-II**) and 2.575 × 10^5^ Da (**PBPC-Ib**) (Li et al., 2017a).

### 3.2 Monosaccharide Composition

The composition of monosaccharides was mostly determined using the 1-phenyl-3-methyl5-pyrazolonde (PMP)-derivatization method or the derivatization with saccharin acetyl (Guo et al., 2021). Both methods begin with acid hydrolysis, followed by derivatization, and finally analysis of the derivatized product. The products of the PMP derivatization method are detected by HPLC, while the saccharin acetyl method yields adducts that are detected by GC (Fan et al., 2013). Because each polysaccharide has a different monosaccharide composition or molar ratio, the structures of polysaccharides are diverse. The heteropolysaccharides, isolated the flowers of safflower, mainly consist of rhamnose, arabinose, mannose, xylose, glucose, and galactose with different molar ratios. Among the SPS fractions, there were eight heteropolysaccharides with different monosaccharide compositions and molar ratios, as well as two homopolysaccharides containing glucose. Notably, the **SPS2**, **SPS3, PBPC-II** and **APBPC-2** fractions, which were isolated from safflower and bee pollen of safflower contain uronic acid. The contents of uronic acid were in the order of 7.06, 6.84 and 11.78%, while the content of uronic acid in **PBPC-II** was not mentioned **(** Zou, 2011; Wang et al., 2019b; Shi, 2021). The differences of monosaccharide composition among these polysaccharides may be caused by different sources of raw materials or different separation and purification methods.

### 3.3 Chemical Structures

Numerous technical methods have been used to demonstrate the structural features of polysaccharides. However, studies on the structural identification of SPS and PBPC are still rare in the literature, and only nine obtained polysaccharides were structurally characterized in detail. The structural characteristics of these nine polysaccharides are listed in details below. The main chain of **CTLP-1** and **CTLP-2** could be determined by periodate oxidation, Fourier transform infrared (FT-IR) spectroscopy, and nuclear magnetic resonance (NMR) spectroscopy (Hu, 2020). The main chain of **CTLP-1** was composed of →1)-GalA*p*-(1→4)-Ara*p*-(1→2)-Glc*p*-(4→, and the main chain of **CTLP-2** was composed of →1)-Gal*p*-(2,6→1)-Ara*p*-(4,6 →1)-Glu*p*-(3→ repeats (Hu, 2020). According to the results of FT-IR spectroscopy, **CTLP-1**, **CTLP-2** and **SPS3** were all composed of a-D-pyranose units, while **SPS2** was a β-D-(1→3)-glucan (Ren et al., 2013; Hu, 2020). In addition, Congo red staining showed that both **CTLP-2** and **SPS2** had a multi-stranded helical structure, which may have a great influence on their biological activity (Ren et al., 2013; Hu, 2020). Based on the results of periodate oxidation, Smith degradation and methylation, the main chain of **CTP** was deduced to be composed of (1→4)-linked glucose and five side chains (Huo et al., 2005b). The backbone structure of **HH1-1**, a neutral arabinogalactan, was found to consist of 1,6-linked galactopyranosyl residues with branched chains at C-3 according to NMR and methylation analysis (Yao et al., 2018). **SPSa** and **SPAW** are both homogeneous polysaccharides composed of β-Glc. However, the glycosidic bonds of their main chains are different, in the order of 1,4,6-β-Glc*p* and (1→3)-linked β-D-Glc*p* (Cui et al., 2019; 2020). According to the FT-IR spectroscopy, β-elimination and iodine reaction, it is presumed that **PBPC-Ⅱ** is a β-pyranosyl polymer with uronic acid units and O-glycosidic bonds, and its main chain may be composed of galactose with long side chains and branches (Wang et al., 2019b).

## 4 Biological Activities

**FIGURE 3:**
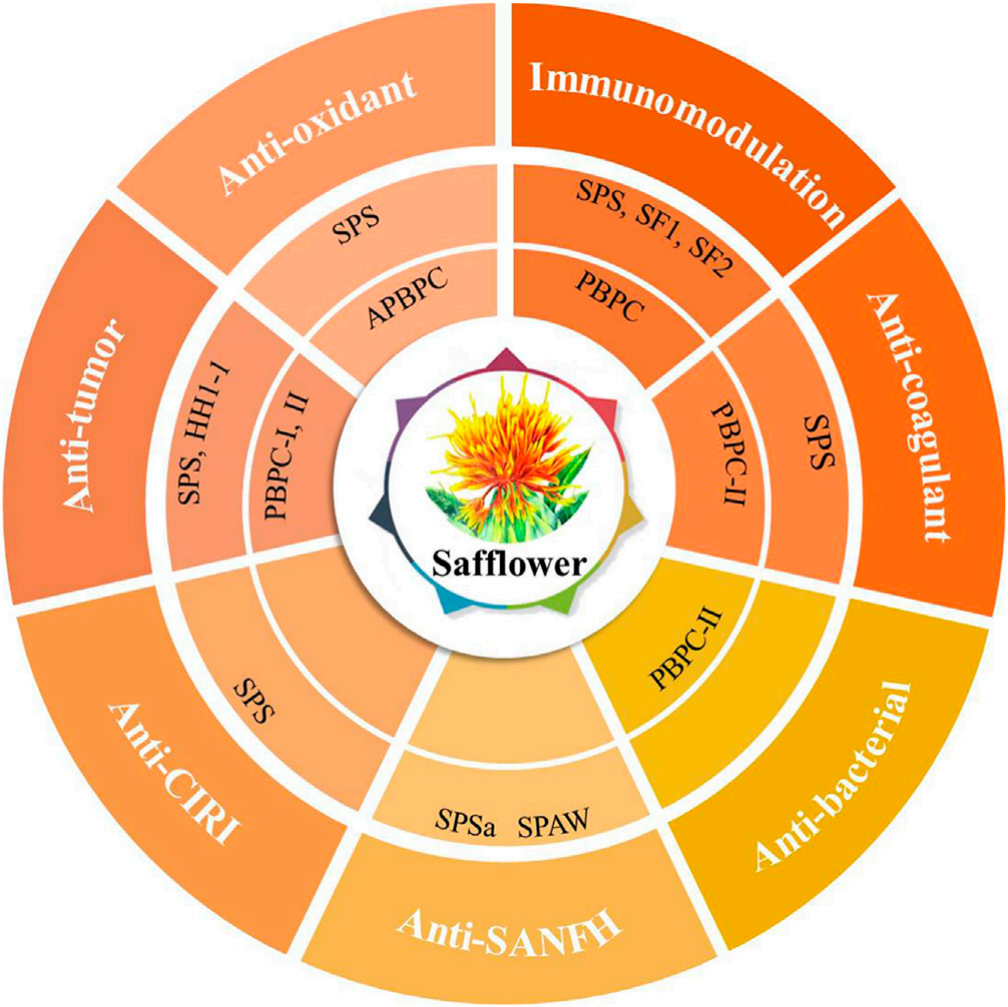
Polysaccharides of safflower and their corresponding bioactivities (CIRI: cerebral ischemia-reperfusion injury; SANFH: Steroid-induced avascular necrosis of the femoral head; SPS: Safflower polysaccharides; PBPC: Polysaccharides of safflower bee pollen; APBPC: Polysaccharide of safflower bee pollen by dilute alkali extraction; Polysaccharide monomers including **SPSa**, **SPAW**, **HH1-1**, **PBPC-I**, **PBPC-II**, **SF1**, and **SF2**).

In the theory of Traditional Chinese Medicine (TCM), safflower is considered to remove blood stasis, promote menstruation and alleviate pain (Zhou et al., 2014). Modern pharmacological studies have demonstrated that safflower with its active compounds possesses wide-ranging biological activities (Zhang and Zheng, 2010; Wang et al., 2016; Liu F. et al., 2018; Ye et al., 2020a; Wu et al., 2021). It has been proven that polysaccharides from the flower or bee pollen of safflower have various biological activities. Here, the advancements in the pharmacological investigation of bioactivities and health benefits in SPS and PBPC are summarized and discussed in details. Polysaccharides of safflower and their corresponding bioactivities are shown in Figure 3. The relevant pharmacological studies and their key results are presented in Supplementary Table S1.

### 4.1 Immunomodulatory Activity

Immunomodulation is one of the most important biological activities of SPS and PBPC. Moreover, SPS can also act as a biological response modifier by enhancing antitumor effects through immunomodulation. It is known that the immune system is composed of immune organs, immune cells, and immune active substances, which all play important roles in immune surveillance, defense, and regulation (Wang and Hu, 2008). Thus, the immunomodulatory activity of SPS and PBPC is illustrated from the three aspects of organs, cells and cytokines.

The immune organ index is a preliminary tool for the assessment of immune capability (Wang et al., 2010). SPS have been proved to improve immune function by increasing the immune organ index. Compared with the control, SPS were found to significantly increase the indexes of the thymus and spleen in H22 tumor-bearing mice and thus enhance the immune function of the organism (He et al., 2009). Huang et al. (1984) found that SPS could effectively counteract the immunosuppressive effect of prednisolone injection, resulting in a significant increase in the weight and cell number of the shrunken spleen, indicating a positive immunomodulatory effect. Moreover, PBPC were found to increase the thymus and spleen index of immunosuppressed mice, antagonize the atrophy of the thymus and spleen caused by Cy, and improve non-specific immunity in mice (Wang L. F. et al., 2020).

Bioactive polysaccharides can directly or indirectly interact with the immune system, triggering several cellular/molecular events, that lead to immune system activation (Leung et al., 2006). Natural killer (NK) cells, T-cells, B-cells and macrophages are the main targets that were found to respond to these molecules (Ferreira et al., 2015). The modulatory effects of SPS are mainly reflected in the activation of immune cells, promotion of cell proliferation, and enhancement of cell killing activity. Both **SF1** and **SF2** can induce the proliferation of B-cells and IgM production, and also stimulate NO production by macrophages in different ways (Wakabayashi et al., 1997). By co-culturing human peripheral mononuclear cells (PBMC) with SPS *in vitro*, it was found that SPS could promote the proliferation of PBMC and CD8^+^ T-cells (Tao et al., 2011), as well as increase the killing activity of NK and LAK cells (Zhou et al., 2010). As a biological response modifier, SPS can partially reverse the inhibitory state of NK cells in a T739 mouse model of lung cancer, and significantly enhance the cytotoxicity of splenic CTL cells and NK cells in tumor-bearing mice (Shi et al., 2010b).

**FIGURE 4:**
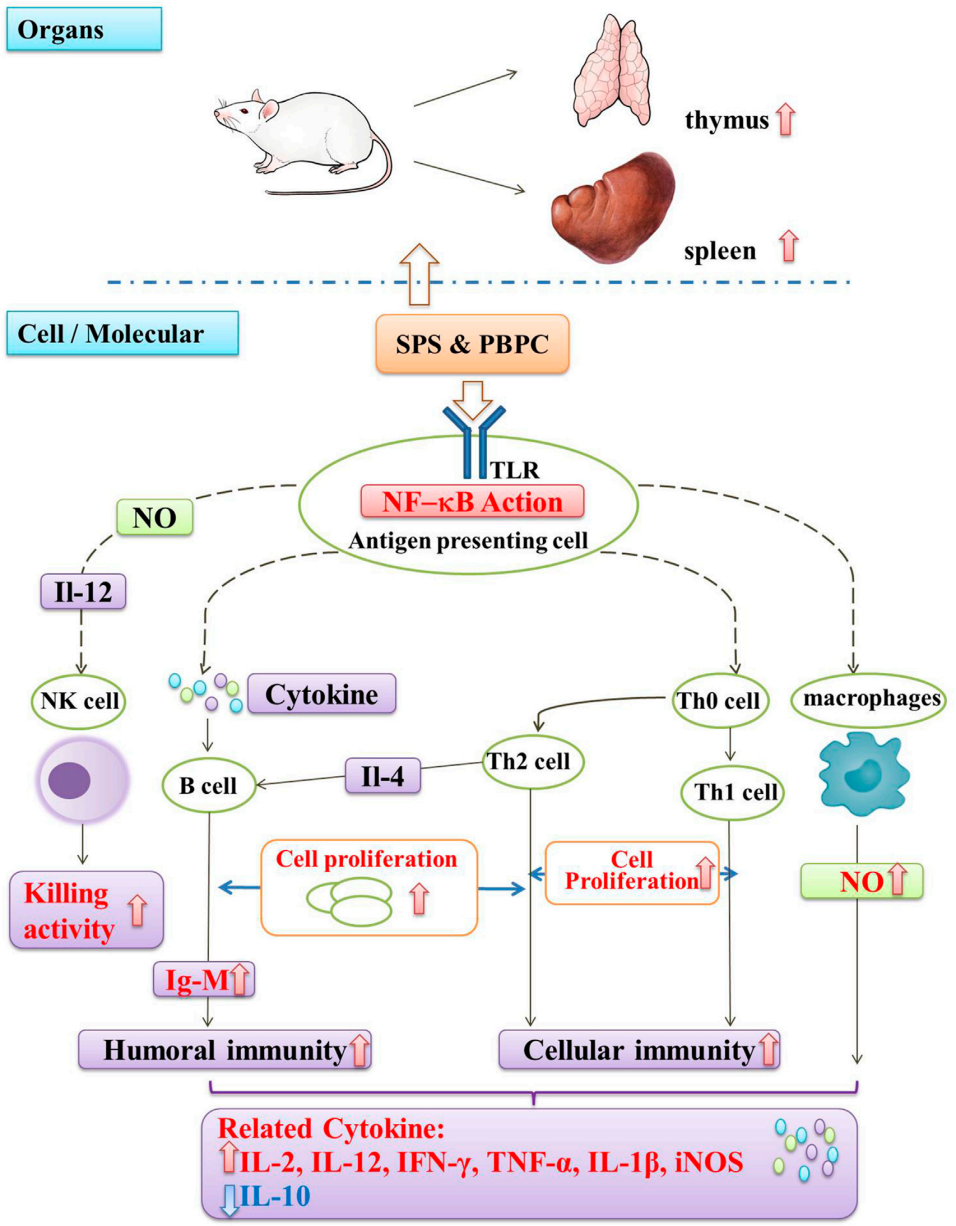
Schematic diagram of immunomodulatory mechanism in SPS and PBPC (Red thick arrow: up-regulate; Blue thick arrow: down-regulate).

Similar to most immunostimulatory polysaccharides, SPS can also enhance the secretion of pro-inflammatory cytokines according to the *in vitro* and *in vivo* experiments. SPS could induce PBMCs to secrete interferon (IFN)-γ and interleukin (IL)-2 (Shi et al., 2010a). In S180 sarcoma mice, Ma et al. found that it could also up-regulate the cytokines IL-12 and tumor necrosis factor (TNF)-α, while down-regulating the cytokine of IL-10 (Ma et al., 2013b). The purified polysaccharides (**HH1-1, SF1, SF2)** obtained from safflower exert their immunomodulatory effects by activating NF-κB signaling. Among them, **HH1-1** increased the expression of the cytokines TNF-α, IL-1β and iNOS in lymphocytes and macrophages (Yao et al., 2018). Similarly, **SF1** and **SF2** induced the production of IL-12 and IFN-γ by peritoneal macrophages in a dose-dependent manner (Ando et al., 2002). It was also reported that PBPC can promote humoral and cellular immunity in mice. Additionally, PBPC were found to promote the ConA-induced transformed proliferation of T lymphocytes in mice (Zuo et al., 2017). A schematic diagram illustrating the immunomodulatory mechanism of SPS and PBPC is shown in Figure 4.

### 4.2 Antioxidant Activity

It is generally believed that polysaccharides exert their antioxidant activity mainly by targeting the NF-κB and Nrf2/Keap1 ARE signaling pathways (Zhang et al., 2019). The intake of polysaccharides can activate pathways and up-regulate the expression of defensive genes, thereby increasing the levels of antioxidant enzymes and molecules such as superoxide dismutase (SOD), glutathione peroxidase (GSH-Px), catalase (CAT), glutathione (GSH), and glutathione reductase (GR), thereby protecting the cells from oxidative stress (Prestera et al., 1995). Reactive oxygen species (ROS) is a general term for oxygen-containing chemicals with strong oxidative capacity, which play an important role in cell signaling and homeostasis *in vivo*. When oxidative stress occurs in the body, the level of ROS will rise sharply, and the oxidative stress product malondialdehyde (MDA) will be produced, which can reflect the degree of oxidative damage (Wang L. et al., 2021; Guo et al., 2021).

The antioxidant activities of SPS and PBPC have been demonstrated in some studies. Wan investigated the antioxidant activity of SPS in H22 tumor-bearing mice, and the results showed that the activities of antioxidant enzymes (GR, GSH-PX, CAT and SOD) were increased, while the ROS and MDA content was decreased in the high-dose group (Wan, 2016). It is suggested that SPS can inhibit ROS production and slow down SOD depletion in the body to a certain extent, thus exerting a protective effect against lipid peroxidation. It was also found that APBPC could increase the activity of SOD and GSH-Px in the serum, liver and brain tissues of a D-galactose-induced mouse model of aging, thereby reducing the levels of the oxidative stress product MDA. This indicates that APBPC can enhance the function of free-radical scavenging, reduce the production of free radicals and exert anti-aging effects (Shi et al., 2020). The purified polysaccharides (**SPS2**, **SPS3**, **CTLP-1**, **CTLP-2**) have also shown antioxidant activity according to the vitro assays (Zou, 2011; Hu, 2020).

### 4.3 Antitumor Activity

**FIGURE 5:**
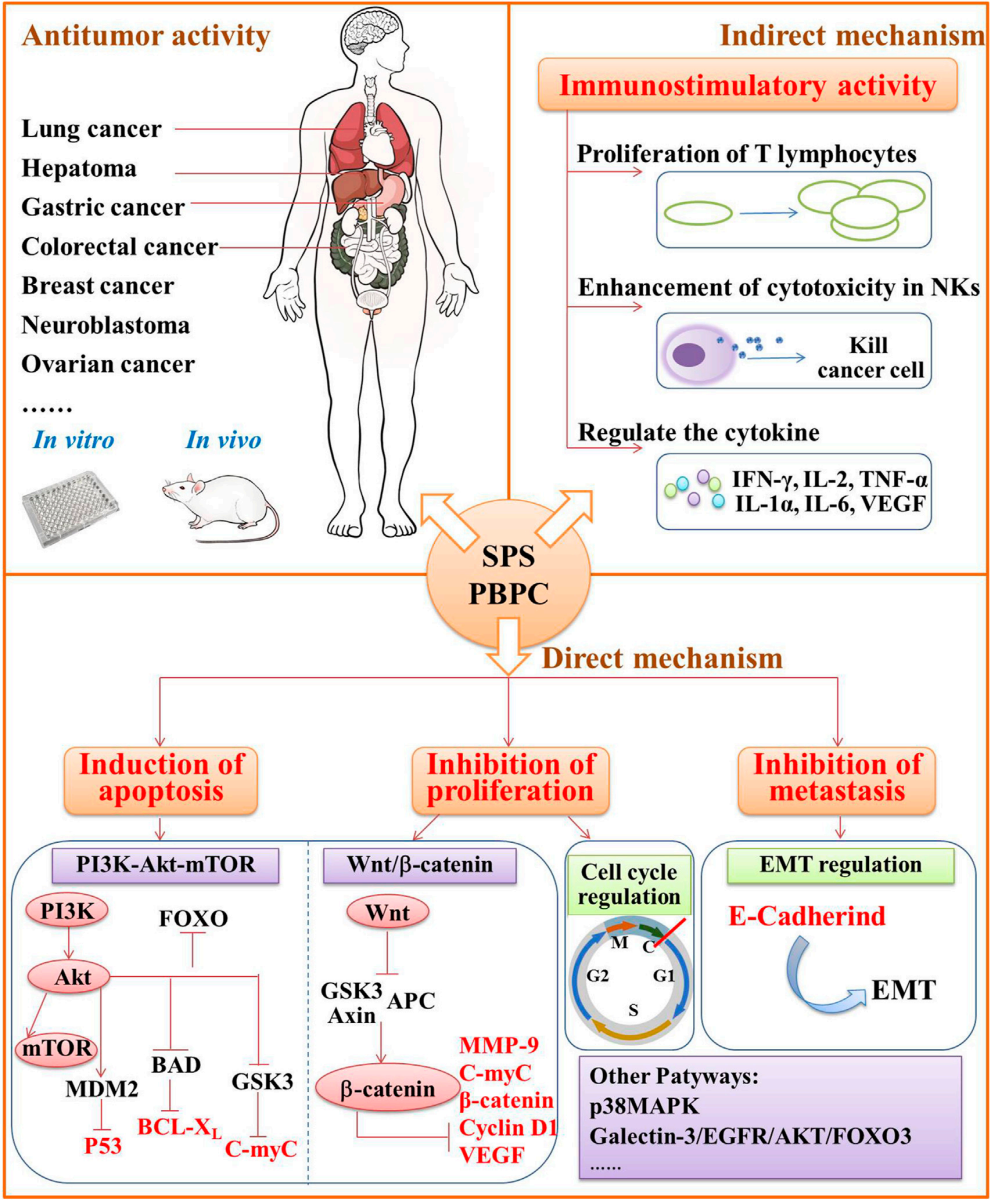
Schematic diagram of antitumor mechanism schematic diagram of in SPS and PBPC.

Malignant tumors remain one of the leading causes of death worldwide (Dong, 2011). Modern pharmacological research has confirmed that the traditional Chinese medicine and its bioactive compounds can exert antitumor effects through multiple pathways and targets (Sun et al., 2021), which has become a new research hotspot in modern oncology. Due to the antitumor effect of safflower and other natural polysaccharides (Zhang et al., 2013; Niu et al., 2017), researchers have conducted a series of in-depth studies on the molecular mechanisms under the anticancer effect of SPS and PBPC. Both classes of polysaccharides have broad-spectrum antitumor activities, and exert their antitumor effects by improving the immune response, inducing the apoptosis of tumor cells and preventing the spread or migration of tumor cells. The detailed antitumor mechanisms of SPS and PBPC are summarized in below, as shown in Figure 5.

#### 4.3.1 Activity Against Gastric Cancer

There are more studies on the effects of SPS on gastric cancer than for other cancers. Apoptosis is one of the main antitumor mechanisms. B-cell lymphoma-2 (Bcl-2), cytochrome C (Cyt-C), caspase-3, Bcl-2-associated X (Bax) and mitochondrial membrane potential (MMP) are considered the key factors for apoptosis (Tsukahara et al., 2006; Li and Lin, 2014; Zhang et al., 2015). *In vitro* experiments revealed that SPS can inhibit the proliferation and induce apoptosis in SGC-7901 human gastric cancer cells by altering the expression of Bcl-2 and Bax at both the mRNA and protein levels, promoting the release of Cyt-C, activating the caspase pathway, and inhibiting MMP (Ma et al., 2012a; Ma et al., 2012b; Ma et al., 2013a; Wang et al., 2015). In addition, SPS also had a certain inhibitory effect on the proliferation of MGC-823 gastric cancer cells and could induce apoptosis (Jiang et al., 2021).

The PI3K/Akt signaling pathway is important for the regulation of cell proliferation. In a variety of malignant tumors, this pathway is over-activated and contributes to the proliferation, migration, invasion and apoptosis of tumor cells (Xu et al., 2021). Thus, SPS can exert anti-cancer effects by inhibiting the PI3K/Akt signaling pathway and inducing apoptosis in human gastric cancer cells. Tao et al. (2012) found that SPS could down-regulate the expression of Akt mRNA in SCG-7901 gastric cancer cells, reduce the expression and phosphorylation of Akt protein, and inhibit the transduction of the PI3K/Akt pathway.

Over-activation of the Wnt/β-catenin pathway is one of the major pathological factors leading to the development and progression of gastric cancer (Wang Q. et al., 2020). Liu et al. demonstrated that inhibition of the Wnt/β-catenin signaling pathway might be a potential antitumor mechanism of SPS. They found that SPS could inhibit the proliferation and invasion of MGC-803 gastric cancer cells, and induce cell cycle arrest at the G0/G1 phase. At the same time, SPS could also inhibit the expression of proteins associated with the Wnt/β-catenin signaling pathway including β-catenin, c-Myc, Cyclin D1, VEGF and MMP-9 (Liu et al., 2020).

#### 4.3.2 Activity Against Lung Cancer

Some scholars have conducted studies on the protective effects of SPS against lung cancer. Interestingly, the results of *in vitro* experiments were inconsistent among different lung cancer cell lines. Zhang et al. (2010b) found that there was no inhibitory effect of SPS on Lewis lung carcinoma cells *in vitro*. However, other experiments showed that SPS could significantly suppress the proliferation of A549 human non-small cell lung cancer cells and induce apoptosis. At an SPS concentration of 0.64 mg/ml, the highest inhibition rate of cell growth reached up to 59% (Dong et al., 2017). Although there was no effect of SPS on Lewis lung carcinoma cells *in vitro*, SPS were found to inhibit tumor growth and metastasis of T739 mice with xenografted Lewis lung carcinoma cells. The weight and volume of the transplanted tumors and metastases were significantly decreased (Zhang et al., 2010b). In-depth research on the relationship of SPS and immune cells was further conducted by Zhang et al. as well as Shi et al. to clarify the underlying mechanisms. Zhang et al. found that SPS promoted the proliferation of T lymphocytes in T739 mice with xenografted Lewis lung carcinoma cells (Zhang et al., 2010b). Shi et al. (2010b) found that the antitumor effects of SPS in lung cancer might be related to enhanced cytotoxicity of NK and CTL cells *in vivo*. All the results mentioned above suggest that SPS may exert its anti-tumor effects by modulating the immune function of the body instead of having a direct cytotoxic effect. The migration and mobility of lung cancer cells was investigated as well. Wang et al. observed the changes in the migration of lung cancer cell lines H460, H1299 and A549 after treatment with SPS. The migration and mobility were effectively inhibited, and the underlying mechanism was found to related to the regulation of genes related to the epithelial-mesenchymal transition (EMT), such as E-Cadherin (Wang, 2016).

#### 4.3.3 Activity Against Liver Cancer

Both SPS and PBPC were found to have antitumor effects against liver cancer. SPS could effectively reduce the tumor volume and nodule growth in rats with liver cancer, and inhibit the proliferation of rat liver cancer CBRH-7919 cells *in vitro* as well (Li, 2017). Liang et al., as well as Sun et al., found that the proliferation of human hepatocarcinoma SMMC-7721 cells was inhibited by SPS, and the mechanism may be related to the expression of cell cycle protein B1 Cdc25B gene or the induction ROS production through the p38MAPK signaling pathway (Liang et al., 2011; Sun et al., 2013; Sun et al., 2014). In addition, SPS was found to affect the Bcl-2/Bax ratio and decrease the mitochondrial membrane potential, thus inducing apoptosis in SMMC-7721 cells (Zhang et al., 2012).

**PBPC-I** was found to have an anti-tumor effect in H22-tumor-bearing mice, which were inoculated with liver cancer cells, by affecting immune function. In detail, **PBPC-I** significantly increased the body mass, spleen index and thymus index of H22-tumor-bearing mice, regulated the serum levels of IFN-γ, IL-2, TNF-α, IL-1α, IL-6, and VEGF, leading to the release of tumor-killing cytokines, and thus inhibited tumor growth and promoted tumor cell apoptosis (Chen et al., 2019).

#### 4.3.4 Activity Against Colorectal Cancer

SPS exerts its activity against colorectal cancer mainly through direct action on tumor cells and indirect effects on the immune system. Sun et al. found that SPS could significantly induce apoptosis and inhibit the growth and invasion of LoVo human colon cancer cells. The effect may be related to Bax, Bcl-2, and caspase-3 (Sun et al., 2016). After SPS treatment, the proliferation of HT29 colon cancer cells was significantly inhibited, and the apoptosis was induced by blocking the cell cycle at the G2/M phase, S phase, as well as up regulating caspase-3 protein expression (Ai et al., 2019). Additionally, the combined application of SPS and NK cells had a synergistic effect on the killing of colon cancer cells *in vitro*. The promotion of cytokines secretion in NK cells influenced by SPS and the increase of their expression levels in NKG2DLs combined with receptor activation might be the relevant mechanisms. Compared to SPS alone, the combined application could enhance the killing sensitivity and clearance of colon cancer cells (Wei et al., 2020).

#### 4.3.5 Activity Against Breast Cancer

Breast cancer is the most commonly diagnosed cancer in women following lung cancer, accounting for 22.9% of all types of cancer in women worldwide and ranking second among causes of cancer-related death in women (Fahad, 2019). Recent studies have mainly investigated the effects of SPS and PBPC on the proliferation, apoptosis and metastasis of breast cancer cells. It was demonstrated that SPS could promote apoptosis in MCF-7 breast cancer cells by regulating the expression of Bcl-2 and Bax (Tao, 2012; Luo et al., 2015), and also induce apoptosis by blocking the PI3K/Akt/mTOR pathway in MDA-MB-435 human breast cancer cells (Liu N. et al., 2018). Similarly, Ding et al. found that SPS inhibited the proliferation of MDA-MB-231 human breast cancer cells. Of greater significance, the effect of the combination of SPS and the hedgehog pathway inhibitor cyclopamine on the proliferation and apoptosis of MDA-MB-231 cells were stronger than either SPS or cyclopamine alone (Ding et al., 2017). Moreover, Luo et al. found that SPS might inhibit the invasion and metastasis of MCF-7 breast cancer cells by influencing the expression of MMP-9 and its specific inhibitor TIMP-1, which are closely related to the physiological and pathological processes of tumors (Luo et al., 2015).

The C-myc and P53 genes play an important role in the development of breast cancer. C-myc gene was one of the earliest identified proto-oncogenes and it has a bidirectional regulatory effect on apoptosis (Wang J. et al., 2020). The P53 protein sequentially binds to DNA in a sequence-specific manner to activate the cell cycle checkpoint for activating cellular senescence, apoptosis or autophagy (Lee and Muller, 2010). Wang et al. found the purified **PBPC-II** from the bee pollen of safflower could inhibit the growth of MDA-MB-231 cells and induce apoptosis. The relevant mechanism might be related to the upregulation of P53 and Bax, combined with the downregulation of C-myc and Bcl-2 (Wang et al., 2019c).

#### 4.3.6 Other Antitumor Activities

In addition to the major antitumor activities mentioned above, SPS and PBPC also have effects on other cancers such as neuroblastoma, ovarian cancer, tongue squamous cell carcinoma, cervical cancer, pancreatic cancer, and prostate cancer.

Neuroblastoma is an embryonal malignancy with a high recurrence rate and poor prognosis, accounting for about 15% of all childhood tumor deaths (Yang et al., 2018). In SH-SY5Y neuroblastoma cells, SPS were able to reduce the proliferation, invasion and metastasis by downregulating the expression of MMP-9, which is one of the major protein hydrolases that can induce the degradation of the extracellular matrix, participating in tumor vasculature and lymphangiogenesis, thereby promoting to tumor invasion and metastasis (Kessenbrock et al., 2010; Qu et al., 2020).

The mutations and overexpression of β-catenin is associated with many malignancies, including rectal, lung, breast, ovarian, and endometrial cancers (Morin, 1999). It was reported that the inhibitory effect of SPS on human ovarian cancer cells is related to the regulation of the Wnt/β-catenin signaling pathway. SPS can reduce the β-catenin protein levels in A2780 ovarian epithelial carcinoma cells in a dose-dependently manner, and thereby inhibiting cell proliferation and metastasis (Zeng and Yang, 2017).

The effect of SPS on tongue squamous cell carcinoma was also investigated. Zhou et al. found that SPS could inhibit the proliferations of HN-6 human tongue squamous carcinoma cells and xenograft tumors in nude mice by inducing apoptosis and cell cycle arrest in the G0/G1 phase. The mechanism might be related to the fact that SPS regulates the mRNA and protein expression of cyclooxygenase-2 (down), Bcl-2 (down), Bax (up), and cleaved caspase-3 (up) in tumor cells (Zhou et al., 2018).

Yang et al., as well as Zhang et al. demonstrated that SPS could inhibit the growth and proliferation of HeLa cells, and promote their apoptosis. Under the influence of SPS, the expression of VEGF, Notch1, Notch2 and MMP-9 decreased as well. Based on these results, the relevant molecular mechanism was deduced. In human cervical cancer cells, the inhibition of growth and proliferation by SPS might be related to VEGF and Notch signaling pathways, while the inhibition of metastasis might be related to the reduction of MMP-9 protein expression (Yang et al., 2016; Zhang et al., 2017).

Zhang et al. have found that SPS were able to inhibit activity, invasion and migration of the B16-F10 melanoma cells. SPS exert antitumor activity against melanoma by activating the Notch1 signaling pathway in macrophages to regulate the polarization of macrophages (Zhang et al., 2021).

Yao et al. demonstrated that the purified SPS (**HH1-1**) had a significant antitumor activity against pancreatic cancer, while having little toxicity to normal pancreatic duct epithelial cells and hepatocytes. As a novel galectin-3 inhibitor, **HH1-1** exerts antitumor activity against pancreatic cancer by blocking the galectin-3/EGFR/AKT/FOXO3 signaling pathway (Yao et al., 2019).

Li et al. found that **APBPC-3** could inhibit the proliferation of DU145 prostate cancer cells, and underlying mechanism might be related to the downregulating of the PI3K/Akt signaling proteins PI3K, Akt-1 and Bcl-2, as well the upregulation of PTEN, P53, caspase-3 and Bax (Li S. F. et al., 2021).

### 4.4 Protective Effect Against Cerebral Ischemia-Reperfusion Injury

Cerebral ischemia-reperfusion injury (CIRI) is a complex temporal and spatial cascade of reactive pathophysiological processes that occur during the recovery of brain tissue from ischemia to perfusion (Ren and Tian, 2021), leading to a more severe pathological response than before perfusion. This disease is often associated with the increase of intracellular free radicals, overload of intracellular Ca2^+^, neurotoxicity of excitatory amino acids, overactive inflammatory response and excessive apoptosis (Yuan et al., 2021). SPS were able to significantly improve the neurological deficits as well as reduce the volume ratio and water content of the infarcted area in rats with cerebral ischemia-reperfusion injury. At the same time, the amounts of TNF-α, IL-1β and IL-10 in the brain tissue of modeled rats were also influenced by SPS. It can be concluded that the mechanism of the protective effects of SPS might be related to the inhibition of the synthesis of inflammatory factors (Ren et al., 2016). Apoptosis of neuronal cells in the ischemic area is one of the most important causes of CIRI. SPS can inhibit the expression of caspase-3 and RAPP in a dose-dependently manner, thus exerting anti-apoptotic effects on neuronal cells for the prevention of CIRI (Wang, 2011).

### 4.5 Protective Effect Against Steroid-Induced Avascular Necrosis of the Femoral Head

Steroid-induced avascular necrosis of the femoral head (SANFH) is a class of non-traumatic femoral head injury that can trigger osteoclast and bone marrow necrosis due to local blood circulation disorders (Wu et al., 2019). The precise mechanism of SANFH remains unclear and may be associated with various factors such as apoptosis and inflammation (Ye et al., 2019). *In vitro* experiment has shown that SPS can inhibit caspase-3 activation in the osteoblasts of rats and promote bone formation (Cui et al., 2018). Cui et al. also investigated the effects of two purified SPS (**SPAW** and **SPSa**) on femoral head necrosis induced by dexamethasone using *in vivo* assays. It was found that the abnormal histopathological changes in the treated group were significantly improved, and the HOM/HOP ratio, which reflects the metabolism of femoral head growth, was increased (Cui et al., 2019; Cui et al., 2020). **SPAW** was also able to significantly down-regulate the expression of Bax and caspase-3 proteins, while upregulating the expression of Bcl-2 protein at the same time. This implies that SPAW exerts an antiapoptotic effect in osteoclasts following SANFH treatment (Cui et al., 2020).

### Other Bioactivities

In addition to the above-mentioned activities, there are other biological activities observed in SPS and PBPC, such as anticoagulant and antibacterial effects. SPS was able to inhibit platelet aggregation induced by 4,5′-adenosine diphosphate disodium salt (ADP) in a concentration-dependent manner. The aggregation inhibition rate was higher than that of the aqueous and alcoholic extracts of safflower (Zhou et al., 2008). The bee pollen polysaccharide fraction **PBPC-II** exerted significant anticoagulant effects *in vitro* by mediating exogenous and endogenous coagulation pathways. It prolonged the activated partial thromboplastin time (APTT) and prothrombin time (PT) in human plasma as well. Compared with the anticoagulant heparin sodium, **PBPC-II** was more effective in prolonging PT (Li et al., 2017a). Moreover, **PBPC-II** was reported to have certain antibacterial effects on *Escherichia coli* and *Staphylococcus aureus* (Li et al., 2017a).

## 5 Structure-Function Analysis

The biological activities of polysaccharides are strongly related to their chemical compositions and configurations (Ye et al., 2020b). Although the relationships between structure and bioactivity have been studied in the natural polysaccharides of some plants, few studies focused on the structure-function relationship of safflower polysaccharides. Here, the relationship can be inferred as follows based on the reported studies (Ren L. et al., 2012; Yu et al., 2018; Wang J. T. et al., 2019; Lu et al., 2020; Wang Q. W. et al., 2021; Liu et al., 2021; Yang et al., 2021). Through a literature review, it can be deduced that the monosaccharide composition and ratio, Mw, and glycosidic bond of safflower polysaccharides are closely related to their antioxidant, immunomodulatory, and antitumor activities.

Arabinose (Ara), ribose (Rib), mannose (Man) and glucose (Glc) were positively correlated with antioxidant activity, whereby glucose had the greatest effect. Conversely, fructose (Fru) and galactose (Gal) were negatively correlated with antioxidant activity (Yang et al., 2021). Polysaccharides with β-(1→4), α-(1→4) or α-(1→6) glycosidic bonds were more likely to have antioxidant activity (Liu et al., 2021). The influence of Mw on antioxidant activity was dependent on the plant sources. Among *Polygonatum odoratum* polysaccharides, fractions with a lower *Mw* were more likely to have stronger antioxidant activity (Yu et al., 2018). However, opposite results were obtained for *Ganoderma lucidum* polysaccharides (Wang Q. W. et al., 2021). By investigating the relationship between the structure and antioxidant activity of safflower polysaccharides, we found that both **SPS2** and **SPS3** have antioxidant activity *in vitro*, whereby **SPS2** is stronger than **SPS3**. In agreement with this finding, both fractions contain monosaccharides (Ara, Man, and Glc) that are positively correlated with antioxidant activity, the *Mw* of **SPS2** (9.332 × 10^3^) is higher than that of **SPS3** (5.861 × 10^3^), and their conformations are different (**SPS2**: β conformation, **SPS3**: α conformation). **CTLP-1** and **CTLP-2** also have antioxidant activity *in vitro*, but their hydroxyl radical scavenging activity is lower than that of **SPS2**, which may be related to their low Mw and α-configuration (Hu, 2020).

Polysaccharides with immunomodulatory activity mostly contain Glc and Man monomers (Liu et al., 2021). Additionally, polysaccharides containing β-(1→4) glycosidic bonds or a main chain structure composed of β-D-(1→3)-Glc repeats are more likely to have immunomodulatory activity (Wang J. T. et al., 2019; Lu et al., 2020; Liu et al., 2021). **SF1** was found to effectively stimulate macrophages to produce various cytokines and thereby exert immunomodulatory effects, while **SF2** had a relatively weak effect (Wakabayashi et al., 1997). Because their glycosidic bond types and specific structures are not clear, the difference in immunomodulatory effects between these polysaccharides may be related to the ratio of Glc.

It was found that D-mannose could influence regulatory T cells and help immune cells recognize and engulf tumor cells (Wang Q. W. et al., 2021). The antitumor activity of polysaccharides is affected by the size of the molecules and their solubility in water. Generally, polysaccharides with a higher Mw have better water solubility and stronger antitumor activity (Ren L. et al., 2012). Due to the insufficient amounts of pure polysaccharides obtained with current isolation and purification methods, researchers often use the crude polysaccharide mixtures for the study of antitumor effects. Only four purified polysaccharides, including **HH1-1**, **PBPC-I**, **PBPC-II**, and **APBPC-3**, were reported to have antitumor effects. Although we found that their Mw was relatively higher than that of other SPS or PBPC fractions, it does not effectively summarize the relationship between the glycosidic conformation of SPS or PBPC and their antitumor effects.

## 6 Conclusion and Future Perspectives

Polysaccharides can be effectively isolated and purified by various methods from different parts of safflower. This paper systematically overviewed the extraction and isolation methods of SPS and PBPC, and summarized their structural characteristics and broad bioactivities including immunomodulatory activity, antioxidant activity, antitumor activity, protective effect against cerebral ischemia-reperfusion injury and steroid-induced avascular necrosis of the femoral head, and others. Like most natural polysaccharides, safflower polysaccharides have immunomodulatory activity. Thus, they can be developed into medicines or health foods for treating the immune-related diseases, or improving the immunity of sub-healthy people. Antitumor effect is another significant activity of safflower polysaccharides, which can be effective for various cancers. They exert antitumor effects through directly effects on tumor cells or indirectly interact with the immune system. Among various extraction methods, ultrasound-assistant extraction and hot water extraction are more efficient for SPS and PBPC, respectively. In spite of the extensive chemical and pharmacological studies of safflower polysaccharides in the past 2 decades, the structural elucidation, especially of high-order structural features, is still a challenge. Additionally, the pharmacological research on pure polysaccharides is limited because of their low content. Due to these challenges, the structure-function analysis has also not progressed to a sufficient degree. Hence, further research should focus on the exact higher-order structures and the structure-bioactivity relationships of safflower polysaccharides, which may offer a comprehensive basis for development of safflower polysaccharides and to deepen our understanding of the effects of natural macromolecules. Based on the significant pharmacological effects of SPS and PBPC, the in-depth pharmacological studies of purified polysaccharide, as well as the identification of specific targets and pathways should be conducted in the future, which will be much more important in fields of new drug discovery and clinical application.
