# Supplementary material for: Extraction, Structures, Bioactivities and Structure-Function Analysis of the Polysaccharides From Safflower (Carthamus tinctorius L.)
Source: Front Pharmacol. 2021 Oct 20;12:767947. doi: 10.3389/fphar.2021.767947 (PMC8563581; doi:10.3389/fphar.2021.767947)
Supplement: Supplementary file 1 [file Table1.docx]

**TABLE S1**

**The pharmacological studies and the key results of SPS and PBPC**

| **NO.** | **Activity** | **Compound (Extract)** | **Model** | **Administration**  **Method** | **Does** | **Function** | **References** |
| --- | --- | --- | --- | --- | --- | --- | --- |
| 1 | Immunomodulation | SPS | PBMC | *In vitro* | 0.312, **0.625**, **1.25**, 2.5, 5 g/L | Promote the proliferation of immune cells;  Promotion of immune active factors production | Shi et al. (2010a); Zhou et al. (2010); Tao et al. (2011) |
| 2 | Immunomodulation | SPS | C_57_ pure line mice;  ICR pure line mice | *In vivo*, *i.p.*;  *In vitro* | 15, 45, 135mg/kg;  125, 250, 500 r/hole | Increase the number of splenocytes to sheep erythrocyte vacuole-forming cells;  Anti-immunosuppressive effect of prednisone;  Promote lymphocyte transformation | Huang et al. (1984) |
| 3 | Immunomodulation | HH1-1 | Splenocytes from BALB/c mice | *In vitro* | 3.53, 7.05,  14.10 μM | Activate NF-кB;  Promote the production of immune active substances | Yao et al. (2018) |
| 4 | Immunomodulation | SF1,SF2 | Splenocytes from C57BL/6 mice; peritoneal exudate cells | *In vitro* | 10^-4^-10^3^ μg/mL | Promote the proliferation of immune cells;  Promote the production of immune active substances;  Activate NF-κB signaling pathway | Wakabayashi et al. (1997); Ando et al. (2002) |
| 5 | Immunomodulation | PBPC | Mice model of cyclophosphamide-induced immune suppression | *In vivo*, *p.o.* | 75, 150, 300 mg/kg | Enhancement of non-specific immunity, humoral immunity and cellular immunity in model mice | Zuo et al. (2017) |
| 6 | Antioxidant | SPS | DPPH; hydroxyl-radical;  copper ion reaction | *In vitro* | 3, 4.5, 6, 7.5, 9, 12, 15 μg/mL | Scavenging of DPPH radicals, hydroxyl radicals;  Reduction of copper ions | Ren et al. (2018) |
| 7 | Antioxidant | SPS2, SPS3 | Hydroxyl radical, superoxide anion | *In vitro* | 100, 200, 300, 400, 500μg/mL | Scavenging of hydroxyl radical;  Superoxide anion | Zou (2011) |
| 8 | Antioxidant | CTLP-1  CTLP-2 | DPPH,  ATBS,  hydroxyl adicals, superoxide anion | *In vitro* | 1, 2, 3, 4, 5mg/mL | Scavenging of DPPH radicals, hydroxyl radicals, ATBS radicals and superoxide anion;  Having Fe^2+^ chelating activity | Hu (2020) |
| 9 | Antioxidant | PBPC | Hydroxyl radical, superoxide anion | *In vitro* | 0.1, 0.2, 0.3, 0.4, 0.5, 0.6 mg/mL | Scavenging of hydroxyl radical, and superoxide anion | Zuo and Qian (2012) |
| 10 | Antioxidant | PBPC-II | DPPH,  hydroxyl radical, superoxide anion | *In vitro* | 0.1, 0.2, 0.3, 0.4, 0.5 mg/mL | Scavenging of DPPH radicals, hydroxyl radicals and superoxide anion | Wang J. T. et al. (2019) |
| 11 | Antioxidant | APBPC | D-Galactose-induced sub-acute aged model in mice | *In vivo*, *p.o.* | 100, 200, 400 mg/kg | Increase the activity of antioxidant enzymes;  Down-regulate the content of MDA | Shi et al. (2020) |
| 12 | Antitumor | SPS | CAM | *In vitro* | 2, 4, **8** g/L | Inhibition of the number and area of CAM angiogenesis | Liang and Wang (2014) |
| 13 | Antitumor | SPS | BALB/c mice bearing S180 | *In vivo*, *i.p.* | 20, 40, 80 mg/(kg·d) | Inhibition of tumor metastasis via decreasing the expression of CD44 and AMF mRNA | Zhao et al. (2014) |
| 14 | Antitumor | SPS | A549 cells of human non-small cell lung cancer | *In vitro* | 0. 04, 0. 08, 0. 16, 0. 32, **0. 64**, 1. 28, 2.56 mg/mL | Inhibit the proliferation of A549 cells;  Promote apoptosis of A549 cells | Dong et al. (2017) |
| 15 | Antitumor | SPS | H460, H1299, A549 cells of lung cancer | *In vitro*, | 1, 2.5, 5, 10, 20 μg/mL | Inhibits the migration and metastasis of lung cancer cells | Wang (2016) |
| 16 | Antitumor | HH1-1 | Eight types of pancancer cells ;  BxPC-3 tumor-bearing mice;  PDX tumors model | *In vitro*;  *In vivo*, *p.o* | 14.10 μM;  0.5,5,50 mg/kg | Inhibit the growth of tumor;  Inhibit the proliferation, migration and invasion of and tumor cell;  Inhibit Galectin-3/EGFR/AKT/FOXO3 signaling;  Inhibit angiogenesis | Yao et al. (2019) |
| 17 | Antitumor | SPS | S180 tumor-bearing mice;  LA795 in T739 mice | *In vivo*, *i.p.* | 10, 20, **40**, 80, 160 mg/kg | Inhibit the growth of tumor;  Increase activity of immune cells in tumor-bearing mice | Shi et al. (2010b) |
| 18 | Antitumor | SPS | Liver cancer model of rat;  CBRH-7919 cells | *In vivo*, *i.g.*;  *In vitro*, | 15, 45, **135** mg/kg;  0.32, **0.64**, 1.28 mg/mL | Inhibit the proliferation of hepatoma cells and tumors | Li (2017) |
| 19 | Antitumor | SPS | Liver cancer cells (SMMC-7721) | *In vitro*, | 0.02, 0.04，0.08, 0.16, 0.32, **0. 64**, 1. 28 g/L | Inhibit the proliferation of SMMC-7721 cells;  Promote apoptosis of SMMC-7721 cells;  Activate P38MAPK signaling pathway | Liang et al. (2011); Zhang et al. (2012); Sun et al. (2013); Sun et al. (2014); Sun et al. (2021) |
| 20 | Antitumor | SPS | H22 tumor-bearing mice | *In vivo*, *i.g.* | 15, 45, 135 mg/kg | Improve immune function;  Increase activity of antioxidant enzymes;  Down-regulation expression of VEGF and Ki67 in mice | He et al. (2009); Wan (2016) |
| 21 | Antitumor | PBPC-I | H22 tumor-bearing mice | *In vivo*, *i.g.* | 100, 200, 400 mg/(kg·d) | Increase the function of immune organ;  Inhibit the growth of tumor;  Promote apoptosis of tumor cell | Chen (2019); Chen et al. (2019) |
| 22 | Antitumor | SPS | HeLa | *In vitro*, | 0.16, 0.32，0. 64 g/L | Inhibit the proliferation of HeLa; Promote the apoptosis and metastasis of HeLa;  Inhibit the PI3K/AKT pathway | Yang et al. (2016); Zhang et al. (2017); Yang et al. (2018) |
| 23 | Antitumor | SPS | Colon cancer cells SW480 | *In vitro*, | 0.315, **0. 625,** 1.25, 2.5 mg /ml | Promote the killing activity of NK cells | Wei et al. (2020) |
| 24 | Antitumor | SPS | LoVo cells | *In vitro*, | 0.5, 1.0, 1.5 g/L | Inhibit the proliferation and invasion of LoVo cells;  Promote the apoptosis of LoVo cells;  Regulates the cell cycle of LoVo cells | Sun et al. (2016) |
| 25 | Antitumor | SPS | HT29 cells | *In vitro*, | 0.02, 0.04, 0.08, 0.16, 0.32, 0.64 g/L | Inhibit the proliferation of HT29;  Promote the apoptosis of HT29 | Ai et al. (2019) |
| 26 | Antitumor | SPS | A2780 cells | *In vitro*, | 0, 0.04, 0.08, 0.16, 0.32, **0.64**, 1.28 g/L | Inhibit proliferation and metastasis of A2780 cells | Zeng and Yang (2017) |
| 27 | Antitumor | SPS | MDA-MB-435 cells | *In vitro* | 0.5, **1.0** mg/mL | Inhibit the proliferation of MDA-MB-435 cells;  Promote the apoptosis of MDA-MB-435 cells;  Inhibition of PI3K/Akt/mTOR pathway | Liu et al. (2018) |
| 28 | Antitumor | SPS | MCF-7 cells | *In vitro* | 0.04, 0.08, 0.17, 0.34, 0.68 or 1.36 mg/ml | Inhibit the proliferation and metastasis of MCF-7 cells;  Promote the apoptosis of MCF-7 cells | Tao (2012); Luo et al. (2015) |
| 29 | Antitumor | SPS | MCF-7 cells;  MCF-7 nude mice transplantation tumor model | *In vitro*;  *In vivo*, *i.g.* | 0.06, 0.12, 0.25, 0.5, **1**, 2. mg/ml | Inhibit the growth of transplanted tumors | Luo (2015) |
| 30 | Antitumor | SPS | MDA-MB231 breast cancer cells | *In vitro* | 0.05, 0.1, 0.2, 0.4, **0.8** g/L | Inhibit the proliferation of MDA-MB231 cells;  Promote the apoptosis of MDA-MB231 cells;  The combination of SPS and cyclopamine is more effective than alone | Ding et al. (2017) |
| 31 | Antitumor | PBPC-II | MDA- MB231 cells | *In vitro*, | 0.12, 0.24, 0.48, 0.96 mg/mL | Inhibit the proliferation of MDA-MB231 cells;  Promote the apoptosis of MDA-MB231 cells | Wang et al. (2019b) |
| 32 | Antitumor | APBPC-2 | MDA-MB231,  DU-145 and HepG-2 cells | *In vitro* | 0.582mg/mL;  0.258mg/mL; 0.215 mg/mL | Inhibit the growth, proliferation and migration of tumor cells;  Promote the apoptosis of tumor cells | Shi (2021) |
| 33 | Antitumor | SPS | SGC-7901 gastric carcinoma cells | *In vitro* | 0.02, 0.04, 0.08, 0.16, 0.32, **0.64** mg/mL | Inhibit the proliferation of SGC-7901 cells;  Promote the apoptosis of SGC-7901 cells;  Inhibition of the PI3K/Akt signaling pathway | Ma et al. (2012a); Ma et al. (2012b); Tao et al. (2012); Ma et al. (2013a); Wang et al. (2015) |
| 34 | Antitumor | SPS | SGC-7901 tumor-bearing mice | *In vivo*, *i.g.* | 30g/kg | Inhibit the growth of tumor;  Decrease the expression of Ang-2; Increased the expression of PTEN | Zhou et al. (2015) |
| 35 | Antitumor | SPS | BGC-823 gastric carcinoma cells | *In vitro* | 0.12, 0.24, 0. 48, 0.96, 1.92 mg/mL | Inhibit the proliferation of BGC-823;  Promote the apoptosis of BGC-823 cells | Jiang et al. (2021) |
| 36 | Antitumor | SPS | MGC-803 gastric carcinoma cells | *In vitro* | 0.16, 0.32, 0.64 mg/mL | Inhibit the proliferation and invasion of MGC-803 cells;  Inhibition of Wnt/β-catenin signaling pathway;  Blocking cell cycle in the G0/G1 phase | Liu et al. (2020) |
| 37 | Antitumor | SPS | SH-SY5Y neuroblastoma cells | *In vitro* | 0.16, 0.32, 0.64 g/L | Inhibit the proliferation, invasion and migration of SH-SY5Y cells; Decrease the expression of MMP-9 | Qu et al. (2020) |
| 38 | Antitumor | SPS | The TSCC cell line HN-6;  HN-6 tumor-bearing mice | *In vitro*;  *In vivo* | 0.02, 0.04, 0.08, 0.16, 0.32, **0.64**, 1.28 mg/mL;  injected with 40 mg/kg for 15d | Inhibit the proliferation of HN-6 cells;  Promote the apoptosis of HN-6 cells;  Blocking cell cycle in the G0/G1 phase;  Inhibit the growth of tumor *in vivo* | Zhou et al. (2018) |
| 39 | Antitumor | SPS | S180 sarcoma mice | *In vivo*, *i.p.* | 20, 40, 80 mg/(kg·d) | Inhibit the growth and metastasis of tumor;  Regulation of cellular immune function in mice;  Increase serum IL-12, TNF-α levels and TIMP-1 expression,  decrease IL-10 levels and MMP-9 expression | Ma et al. (2013b); Liang et al. (2017) |
| 40 | Antitumor | PBPC-II | S180 sarcoma mice | *In vivo*, *i.g.* | 100, 200, 400 mg/(kg·d) | Inhibit the growth of tumor; promote the apoptosis tumor cells;  Enhance the immune function of mice | Chu (2019) |
| 41 | Antitumor | APBPC-3 | DU145 prostate cancer cells | *In vitro* | 50, 100, 200, 300, 600 μg/mL | Inhibit the proliferation of DU145 cells;  Inhibition of the PI3K/Akt signaling pathway | Li et al. (2021) |
| 42 | Protective effect of CIRI | SPS | CIRI model in Wistar rats | *In vivo*, *i.g.* | 25, 50, 100 mg/(kg·d) | Inhibit the production of inflammatory factors;  Reduce apoptosis of neural cells | Ren et al. (2016) |
| 43 | Protective effect of SANFH | SPS | Primary osteoblastic cells from Wistar rats | *In vitro* | 25, 50, 100 μg/ml | Promote the proliferation of murine osteoblasts;  Inhibit the apoptosis in osteoblasts; | Cui et al. (2018) |
| 44 | Protective effect of SANFH | SPSa | Rats | *In vivo*, *i.g.* | 25, 100mg/kg | Improve the abnormal histopathological changes;  Inhibit the apoptosis of osteocytes;  Increase bone mineral density and the ratio of HOM/HOP | Cui et al. (2019) |
| 45 | Protective effect of SANFH | SPAW | Rabbits | *In vivo*, *p.o.* | 25, 100, 200 mg/kg | Improve the abnormal histopathological changes;  Inhibit the apoptosis of osteocytes;  Increase the ratio of HOM/HOP | Cui et al. (2020) |
| 46 | Anticoagulant | SPS | Rabbits | *In vitro* | 6, 12, 24, **48** mg/mL | Inhibition of ADP-induced platelet aggregation | Zhou et al. (2008) |
| 47 | Anticoagulant | PBPC-II | Blood | *In vitro* | 10, 50, 100, 150,  200 mg/L | Prolong the clotting time of APTT and PT | Li et al. (2017a) |
| 48 | Antibacterial | PBPC-II | Bacteria | *In vitro* | 8, 6, 4, 2 mg/mL | Inhibit Escherichia coli and Staphylococcus aureus | Li et al. (2017a) |

Note: SANFH: steroid-induced avascular necrosis of the femoral head; CIRI: cerebral ischemia-reperfusion injury; Bold font is the optimal dosage.
